# Promoting oligodendroglial-oriented differentiation of glioma stem cell: a repurposing of quetiapine for the treatment of malignant glioma

**DOI:** 10.18632/oncotarget.16400

**Published:** 2017-03-21

**Authors:** Yun Wang, Nanxin Huang, Hongli Li, Shubao Liu, Xianjun Chen, Shichang Yu, Nan Wu, Xiu-Wu Bian, Hai-Ying Shen, Chengren Li, Lan Xiao

**Affiliations:** ^1^ Department of Histology and Embryology, Chongqing Key Laboratory of Neurobiology, Third Military Medical University, Chongqing 400038, China; ^2^ Department of Pathology, Southwest Hospital, Chongqing 400038, China; ^3^ Department of Neurosurgery, Southwest Hospital, Chongqing 400038, China; ^4^ Robert Stone Dow Neurobiology Laboratories, Legacy Research Institute, Portland, OR 97232, USA

**Keywords:** quetiapine, glioma stem cell, proliferation, differentiation, Wnt/β-catenin signaling pathway

## Abstract

As a major contributor of chemotherapy resistance and malignant recurrence, glioma stem cells (GSCs) have been proposed as a target for the treatment of gliomas. To evaluate the therapeutic potential of quetiapine (QUE), an atypical antipsychotic, for the treatment of malignant glioma, we established mouse models with GSCs-initiated orthotopic xenograft gliomas and subcutaneous xenograft tumors, using GSCs purified from glioblastoma cell line GL261. We investigated antitumor effects of QUE on xenograft gliomas and its underlying mechanisms on GSCs. Our data demonstrated that (i) QUE monotherapy can effectively suppress GSCs-initiated tumor growth; (ii) QUE has synergistic effects with temozolomide (TMZ) on glioma suppression, and importantly, QUE can effectively suppress TMZ-resistant (or -escaped) tumors generated from GSCs; (iii) mechanistically, the anti-glioma effect of QUE was due to its actions of promoting the differentiation of GSCs into oligodendrocyte (OL)-like cells and its inhibitory effect on the Wnt/β-catenin signaling pathway. Together, our findings suggest an effective approach for anti-gliomagenic treatment via targeting OL-oriented differentiation of GSCs. This also opens a door for repurposing QUE, an FDA approved drug, for the treatment of malignant glioma.

## INTRODUCTION

Malignant glioma accounts for 30% of all brain tumors and 80% of malignant brain tumors. Although tremendous effort has been devoted to the development of treatment approaches, the prognosis of glioma is still poor and has a very low 5-year survival rate [1]. Treatment outcomes are particularly disappointing because of the existence of a subgroup of glioma cells called glioma stem cells (GSCs), which are characterized by self-renewal and multiple differentiation potentials [2, 3]. GSCs are thought to be generated by asymmetric division of neural stem cells (NSCs) or oligodendrocyte progenitor cells (OPCs), as they share the same markers including CD133, Sox2, Nestin, PDGFRβ and others [4–6]. Importantly, GSCs can survive traditional radio- or chemotherapy [7, 8], therefore understanding these cells may contribute to solving the core problem in glioma treatment [9].

Clinically, radiotherapy combined with the alkylating agent temozolomide (TMZ) is the standard treatment that indeed does improve overall survival of glioma patients [10]; however, chemotherapeutic drugs can induce DNA damage and mainly kill fast proliferating tumor cells, not GSCs [11, 12]. Furthermore, due to the characteristic high stemness of these cells, GSCs can give rise to recurrent tumors even after treatment [13]. These features may serve to explain the poor outcomes of current therapies for glioma. Therefore, targeting GSCs may increase sensitivity of glioma to radio- and chemotherapy [14, 15]. Accumulating studies suggest that manipulation of cellular pathways related to generation and stemness of GSCs could be a promising way to inhibit glioma genesis. For instance, inhibiting the Notch signaling pathway by using a r-secretase inhibitor can induce the differentiation of the GSCs into neuron- or astrocyte-like cells and increase the sensitivity of the glioma to TMZ therapy [16, 17]. Conversely, active Notch signaling can enhance the radio- and chemoresistance of GSCs [18, 19]. Likewise, inhibition of the Wnt signaling reduced proliferation and sphere forming capacity of GSCs [20], whereas activation of Wnt signaling is closely associated with radio- and chemoresistance [21, 22]. Recent studies demonstrated that a Wnt antagonist, secreted frizzled-related protein 4 (sFRP4), can reduce GSCs by inducing cell apoptosis or repressing cell proliferation [23]. Moreover, inhibition of the Wnt signaling can chemo-sensitize the GSCs to TMZ [23, 24]. However, no practical medication is currently available.

On the other hand, recent studies suggest that antipsychotic drugs are potential candidates for glioma treatment. It has been shown in epidemiological studies that the incidence of cancer seems to be negatively correlated with the antipsychotics treatment in patients with schizophrenia as compared to normal individuals [25]. It is also known that the antipsychotic drug thioridazine is able to selectively target neoplastic cells and impair cancer stem cells, while having no effect on normal cells [26]. Also, olanzapine, an atypical antipsychotic drug, has been shown to inhibit *in vitro* proliferation and migration of glioblastoma cells and enhance the anti-tumor activity of TMZ [27]. These studies suggest an antitumor feature of antipsychotic drugs against *in vivo* glioma. To date, there have been few studies regarding the underlying target and/or pathway of antipsychotics on gliomagenesis, which is crucial in the development of glioma treatment. Our recent findings suggest that the differentiation of GSCs may be a targeted process contributing to antipsychotics-related anti-glioma effect [28, 29]. We demonstrated that (i) quetiapine (QUE), an atypical antipsychotic, can induce the differentiation of the NSCs into OLs [28]; (ii) QUE can prevent tumor metastasis by repressing breast cancer induced osteoclast differentiation [29]. Since well differentiated glioma, such as oligodendrogliomas, are more susceptible to chemotherapy than other undifferentiated gliomas [30, 31], we proposed QUE as an anti-gliomagenic treatment by promoting the differentiation of GSCs into OL-like cells and sensitizing them for TMZ treatment.

To test this hypothesis, we first examined the effect of QUE on GSCs in cultured cells purified from glioblastoma cell line GL261. Secondly, we established xenograft gliomas *in vivo* to examine the treatment effect of QUE, as well as its combined application with TMZ. Thirdly, we applied a treatment paradigm of QUE following TMZ withdrawal to test the specific effect of QUE on GSCs. For the first time, we demonstrated that QUE can promote the differentiation of GSCs into OL-like cells through inhibition of the Wnt signaling pathway. As a FDA approved drug, QUE may become a promising candidate for glioma therapy.

## RESULTS

### QUE promotes differentiation of GSCs into oligodendrocyte-like cells

To evaluate the effect of QUE on GSCs, we first isolated cells from the glioblastoma cell line GL261 to form spheres in a serum-free conditional medium with EGF and bFGF (Supplementary Figure 1A). Most of the cells demonstrated positive staining for stem markers CD133 (98.56% ± 1.53%), Sox2 (97.25% ± 2.25%) and Nestin (98.37% ± 1.02%) in proliferation media. After being cultured in differentiation media (containing 10% fetal bovine serum without growth factors) for 7 days, the majority of the cultured cells expressed GFAP (glial fibrillary acidic protein, an astrocyte marker) (88.77% ± 3.42%) whereas few cells expressed MBP (myelin basic protein, an oligodendrocyte marker) (8.34% ± 1.03%) (Supplementary Figure 1B-1D). Moreover, these cells were able to form subcutaneous or orthotopic xenograft tumors with pathological characteristics of gliomas after being implanted into mice for four weeks (Supplementary Figure 1E-1F), indicating successful purification of GSCs in culture.

**Figure 1:**
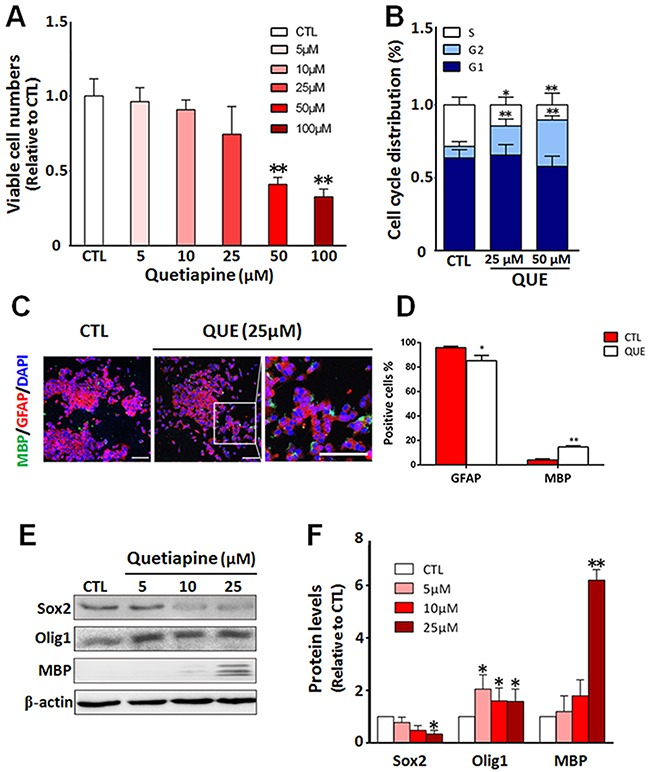
QUE inhibits proliferation but promotes differentiation of GSCs into oligodendrocyte-like cells *in vitro* **(A)** Cell counting assessment showing viable cell numbers after *in vitro* treatment of QUE (5 ~ 100μm) *vs* controls (CTL). **(B)** Cytometry assay showing changes in cell cycle status of GSCs after QUE treatments. **(C)** Representative immunofluorescence staining showing cellular changes of lineage cell markers after QUE treatment: GFAP (red), MBP (green) and nuclei marker DAPI (blue). Scale bar: 100μm. **(D)** Quantitative analysis of GFAP and MBP positive cells after QUE treatment. **(E)** Representative Western blot revealing the expression levels of GSC marker (Sox2) and oligodendroglia lineage markers (Olig1, MBP) in cells with treatments of QUE (5, 10, 25μM) *vs* controls (CTL). **(F)** Quantification of protein levels of Sox2, Olig1 and MBP from GSCs after treatment of QUE (5, 10, 25 μM) *vs* controls (CTL). Data were from three independent experiments. *P <0.05, **P<0.01 *vs* CTL.

To test the effect of QUE on proliferation, we treated GSCs cultured in proliferation media with QUE in different concentrations (0, 5, 10, 25, 50, or 100μM) for designated time periods. The CCK assay results showed a trend of down-regulation of viable cell numbers in correspondence with increasing concentrations of QUE, and this decrease was statistically significant in high dose treatments (50 μM and above) of QUE (Figure 1A). The decrease in viable cells was likely due to a cytotoxic effect. Analysis of the cell cycle indicated that when compared to the control, the ratio of QUE-treated GSCs in S phase was significantly reduced, whereas the ratio of cells in G2-M phase was increased (Figure 1B); this shift indicates that relatively high doses of QUE (>25 μM) may inhibit cell proliferation by retarding cell cycle in the G2-M phase. Therefore, subsequent experiments were performed using 25μM QUE.

We then further evaluated the effect of QUE on GSC differentiation. GSCs were cultured in differentiation medium with or without QUE treatment. Our immunofluorescence staining revealed that after QUE treatment, the number of MBP positive cells was significantly increased, whereas the number of GFAP positive cells was reduced (Figure 1C). This immunofluorescence result was further confirmed by Western blot assessment; QUE down-regulated the expression of GSC marker Sox2, yet up-regulated OL lineage marker MBP and Olig1 in a dose-dependent manner (Figure 1D–1E). Together, our data indicate that QUE can both promote differentiation of GSCs towards OL-like cells and suppress GSC proliferation *in vitro*.

### QUE inhibits Wnt/β-catenin pathway during GSC differentiation

**Figure 2:**
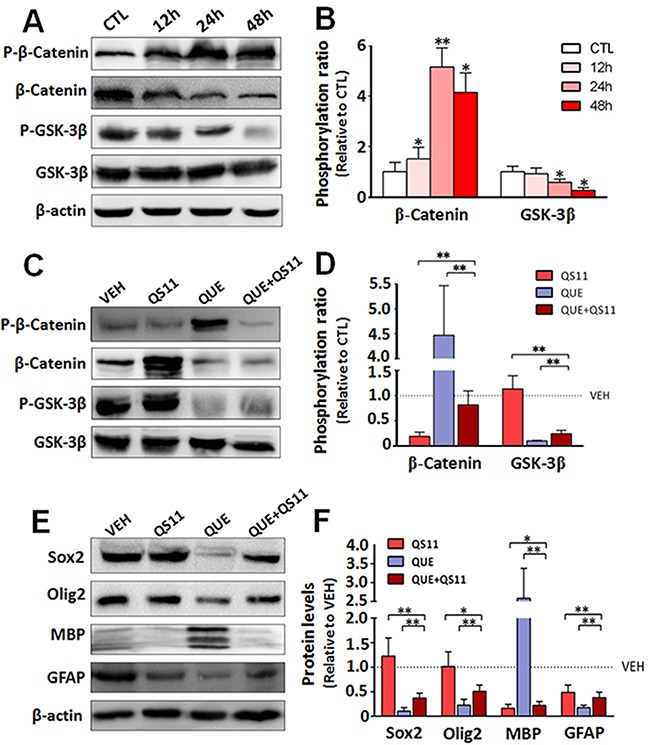
QUE inhibits Wnt/catenin signaling during differentiation of GSCs **(A)** Representative Western blot and **(B)** quantification for the Western blot of the phosphorylation level of β-catenin (p-β-Catenin) and GSK3β (p-GSK3β) *vs* their total protein level (β-Catenin and GSK3β) in GSCs after 12, 24 and 48 h of QUE treatment (% of CTL). **(C)** Representative Western blot and **(D)** Quantification for the Western blot of the expression level of total and phosphorylated β-catenin and GSK-3β in GSCs after treatment of QUE, QS11, or combined QUE+QS11 v*s* VEH (as 100%). **(E)** Representative Western blot and **(F)** Quantification for the Western blot of the expression levels of lineage markers Sox2, Olig2, MBP, and GFAP in GSCs after treatment of QUE, QS11, or combined QUE+QS11 *vs* VEH (as 100%). *P <0.05, **P<0.01 *vs* control **(B)** or between indicated groups **(D, F)**.

To mechanistically investigate the QUE-mediated effect on GSCs, we evaluated the outcome of Wnt signaling, as it has been shown to play an important role in proliferation and differentiation of GSCs [32]. Using the same paradigm of GSC culture and QUE treatment described above, we assessed the activation of Wnt/β-catenin signaling in GSCs during differentiation with or without QUE treatment. Western blot results showed that: (i) after QUE treatment the phosphorylation levels of GSK3β were decreased, whereas phosphorylation levels of β-catenin (P-β-catenin) were increased in a time- and concentration-dependent manner (Figure 2A–2B); (ii) after co-administration of QUE with QS11 (an agonist of Wnt-signaling pathway), QUE-induced reduction of P-GSK3β (Ser9) and increase of P-β-catenin was attenuated by QS11 (Figure 2C–2D). Moreover, QS11 application reversed QUE-induced effects, causing up-regulation of Sox2, Olig2 and GFAP expressions, as well as down-regulation of MBP (Figure 2E–2F). These results indicate that the QUE-induced promotion of GSC differentiation is mediated by its inhibition of the Wnt/β-catenin signaling pathway.

### QUE conducted anti-tumor effect that can be enhanced in combination with TMZ

**Figure 3:**
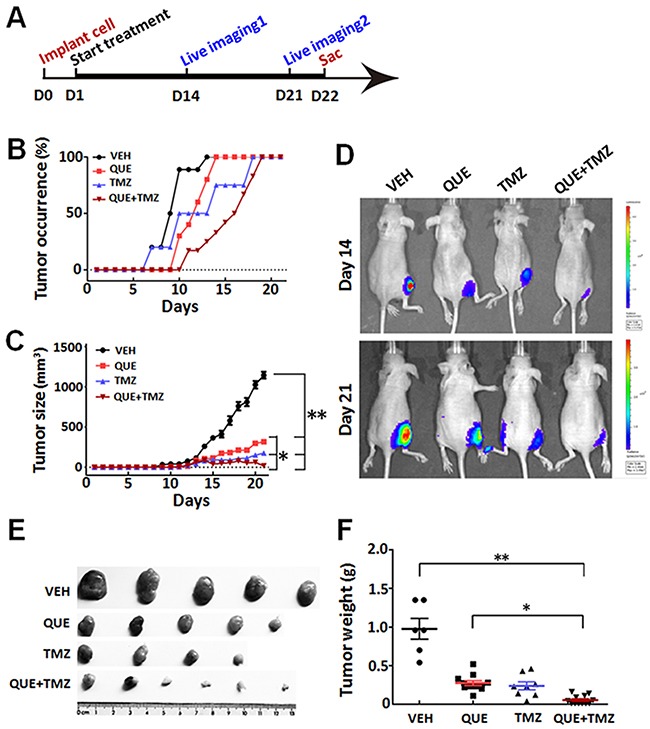
Therapeutic effect of QUE, TMZ, and combined QUE and TMZ on heterotopic xenograft tumors initiated by GSCs **(A)** Experimental paradigm of cell implantation, drug treatment, bioluminescence imaging, and tumor burden analysis on heterotopic xenograft tumor model. **(B)** Ratio of tumor occurrence in mice subcutaneously implanted with GSCs, through the period of treatments of QUE, TMZ, or combined QUE+TMZ *vs* VEH controls up to 21 day (*n*= 6 per group). **(C)** Tumor size after GSC implantation and 21-day treatment of QUE, TMZ, or combined QUE+TMZ (*n*= 6 per group). **(D)** Representative bioluminescence images of mice bearing subcutaneously implanted luciferase-labeled GL-261 glioma cells at day 14 or day 21 after treatments of QUE, TMZ, or combined QUE+TMZ *vs* VEH controls, respectively. **(E)** Representative tumor masses dissected from GSC-implanted mice with treatment of QUE, TMZ, or QUE+TMZ for 21 days. **(F)** Quantitative analysis of tumor mass weight from GSC-implanted mice after 21-day treatment of QUE, TMZ, or QUE+TMZ *vs* controls (VEH). *P < 0.05, **P < 0.01 *vs* VEH, or between indicated groups.

**Figure 4:**
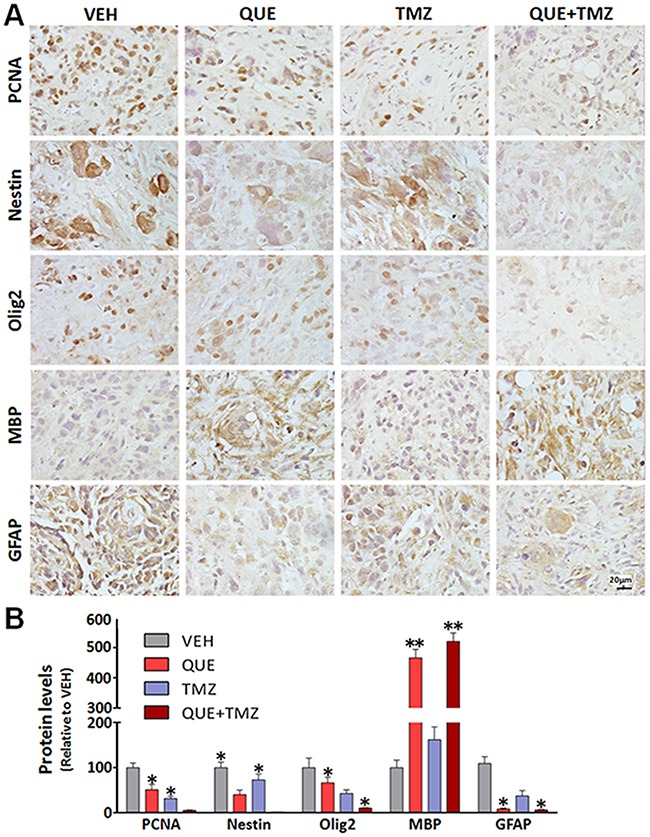
Differentiation changes of GSC-initiated heterotopic gliomas after treatment of QUE, TMZ or combined QUE and TMZ **(A)** Representative immunohistochemistry of PCNA, Nestin, Olig2, MBP, and GFAP in GSC-initiated heterotopic xenograft gliomas after 21-day treatment of QUE, TMZ or combined QUE+TMZ *vs* controls (VEH). **(B)** Quantitative analysis, from photomicrographs, for the immunohistochemistry results of cellular markers in GSC-initiated gliomas after treatment of QUE, TMZ or combined QUE+TMZ for 21 days. *P <0.05. **P<0.01 *vs* VEH. Scale bar: 20μm.

To determine whether QUE can inhibit the growth of GSC-initiated tumors *in vivo*, we established a heterotopic xenograft tumor model in nude mice and an orthotopic xenograft model of glioma in C57 mice. First, mice with subcutaneously GSC-induced tumors were treated with a monotherapy of QUE or TMZ, or combined QUE+TMZ *vs* vehicle controls for 21 days (Figure 3A). In the vehicle-treated control group, mice formed small tumors at the subcutaneous injection site 7 days after injection, and the average diameter of the tumor reached 1.44 ± 0.43 cm at day 21. In contrast, no tumors were found in the treatment groups until day 10 post-GSC implantation (Figure 3B) and the average diameter (0.70 ± 0.03 cm) of the tumors was also smaller than that of the vehicle-treated controls (*p*<0.05) (Figure 3C). Of note, tumor formation was largely suppressed in the combined QUE+TMZ group, indicated by both gross measurement and bioluminescence imaging of the tumors (*p*<0.05) (Figure 3D–3F). Cellularly, histopathological staining showed that treatment of QUE or TMZ reduced the number of positively stained cells for proliferating cell nuclear antigen (PCNA) (Figure 4A), indicating their anti-proliferative effect on tumors. Additionally, Sox2, Olig2 and GFAP were down-regulated while MBP was up-regulated in the QUE treatment group, but not in the TMZ group (Figure 4A). Western blot results further confirmed that QUE significantly up-regulated MBP expression, but down-regulated vimentin (a marker for glioma) and GFAP expression (Figure 4B). These data suggest that QUE but not TMZ may promote differentiation of GSCs towards OL-like lineage *in vivo*.

**Figure 5:**
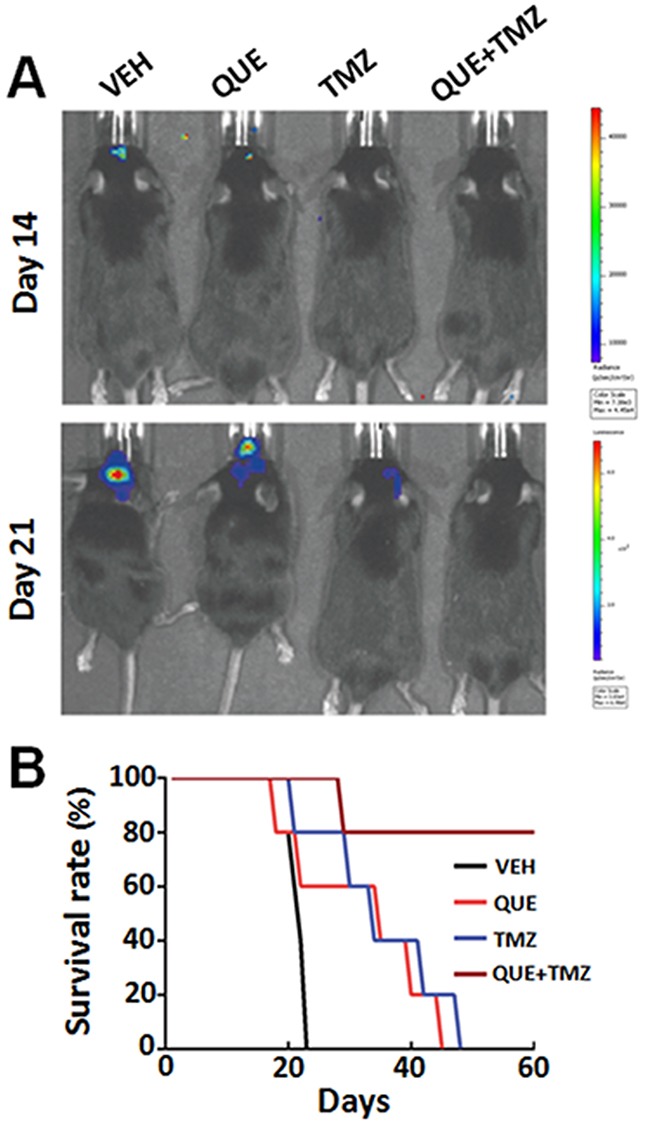
Combined treatment of QUE and TMZ increases overall survival rate of mice with orthotopic GSC-initiated glioblastomas **(A)** Representative bioluminescence images of mice bearing orthotopically implanted luciferase-labeled GL-261 glioma cells after treatment of QUE, TMZ or combined QUE+TMZ, for 14 or 21 days, respectively. **(B)** The Kaplan–Meier plot showing survival rate of mice in different treatment groups (*n* = 10 per group).

To further determine the therapeutic effect of QUE on glioma in a clinically relevant model, we transplanted GSCs intracranially into the striatum to establish an orthotopic xenograft glioma mouse model. On the day of cell implantation, mice began to receive treatments of QUE, TMZ, or QUE+TMZ (see Methods). In line with the findings of the above study using subcutaneous xenograft tumor models, we observed that glioma growth was dramatically suppressed by either monotherapy of QUE or TMZ, or the combined QUE+TMZ treatment (Figure 5A), as evidenced by bioluminescence data of day 14 and day 21 after GSCs transplantation. Among the three treatments, the combined QUE+TMZ treatment had the highest antitumor efficacy (Figure 5A) and the highest survival rate (75% survival at 60 days after glioma establishment). Nonetheless, the survival rate of mice with glioma was increased in all three treatment groups *vs* vehicle controls (Figure 5B). In addition, we have conducted further experimentation, using annexin V/PI staining, to compare apoptosis of GSCs among the treatments of QUE, TMZ, or QUE+TMZ. The annexin V/PI staining showed that compared to the vehicle control, the combined treatment of QUE and TMZ led to significant cell apoptosis (Supplementary Figure 2) whereas treatment with either QUE or TMZ alone was devoid of this pro-apoptosis effect on GSCs. These data indicate that (i) QUE has anti-tumor activity and this activity can be enhanced by combination with TMZ; (ii) apoptosis maybe one of the mechanisms involved in this effect.

### QUE suppresses the growth of TMZ-resistant gliomas initiated by GSCs

**Figure 6:**
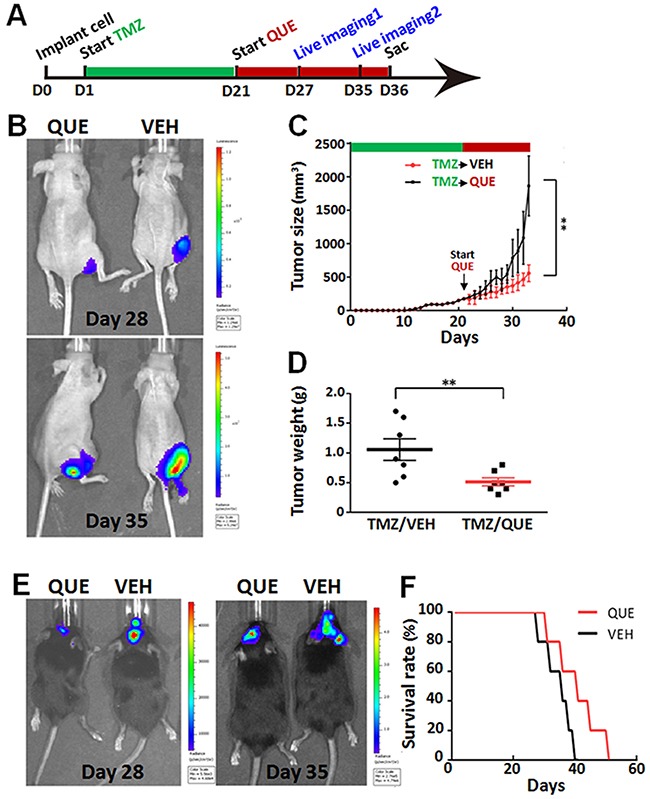
QUE inhibits growth of gliomas initiated by GSCs that are TMZ-tolerant **(A)** Experimental paradigm of drug treatment and bioluminescence imaging in mouse models of heterotopic xenograft tumor or orthotopic xenograft glioma. **(B)** Representative bioluminescence imaging of tumors from mice bearing subcutaneously implanted luciferase-labeled GSCs, after 7-day (Day 28) or 14-day (Day 35) follow-up treatment of QUE *vs* vehicle (VEH). **(C)** Tumor size of tumors from mice after 7- or 14-day follow-up treatment of VEH or QUE after TMZ (*n* = 10 per group). **(D)** Tumor weights from mice after 14-day follow-up treatment of VEH or QUE (TMZ/VEH and TMZ/QUE, *n* = 10 per group). **(E)** Representative bioluminescence tumor imaging from mice bearing orthotopically implanted luciferase-labeled GL-261 glioma cells, after 7-day (Day 28) or 14-day (Day 35) follow-up treatment of QUE or VEH. **(F)** Survival rate of mice with TMZ-tolerant orthotopic xenografted glioma after follow-up treatment of QUE *vs* VEH (*n* = 10 per group).

To further determine if QUE specifically targets the GSCs in gliomas, we established two GSC-related tumor models in mice (subcutaneous xenograft tumor and orthotopic xenograft glioma) combined with an intervention paradigm of TMZ pretreatment followed by QUE treatment. TMZ mainly kills fast proliferating tumor cells, not GSCs; this ensures that QUE's effects, if seen, are caused mainly by effecting GSCs [33] (see Methods and Figure 6A). In both models, tumors were observed 7 days after TMZ withdrawal. However, compared to vehicle-controls, QUE treatment significantly suppressed tumor growth, as determined by size and weight of the tumors (Figure 6C–6D). Bioluminescence data also revealed a similar effect of QUE in both subcutaneous xenograft tumor and orthotopic xenograft glioma models 7 and 14 days after TMZ withdrawal, i.e., at Day 28 and Day 35 in Figure 6B and 6E. Importantly, QUE treatment dramatically increased the survival rate of mice (Figure 6F). These results suggest that QUE can specifically target GSCs and effectively inhibit TMZ- resistant (or –escaped) tumors generated from GSCs.

## DISCUSSION

In the present study, we investigated the therapeutic effects of the antipsychotic quetiapine (QUE) on gliomagenesis and further explored its underlying mechanisms. Using mouse models of GSC-initiated subcutaneous xenograft tumors and orthotopic xenograft gliomas, we demonstrated that: (i) QUE effectively repressed GSCs-initiated tumor growth; (ii) the anti-glioma effect of QUE was due to the combination of its inhibition of the Wnt/β-catenin signaling pathway along with its promotive action on differentiating GSCs into OL-like cells; (iii) QUE demonstrated synergistic antitumor effects with TMZ on gliomas as compared to either individual monotherapy; and importantly (iv) QUE can specifically inhibit GSCs in gliomagenesis.

Previous epidemic studies revealed that patients with schizophrenia were less likely to be troubled by glioma [34]. Despite this knowledge and its common usage as a prescribed drug for patients with schizophrenia, QUE has never been evaluated for its effects on glioma; our evaluation of QUE-mediated effects on GSC-initiated glioblastoma was intended to help explain the phenomenon observed. GSCs are the major source for gliomagenesis, and may originate from the transformation of stem or progenitor cells, including NSCs or OPCs. In addition, GSCs may also result from de-differentiation of mature neurons, OLs, or astrocytes [35]. Those cells are responsible for chemo-resistance and the initiation of recurrent tumors, and are thus considered an essential target in glioma treatment [33]. In this study, we demonstrated for the first time that QUE can inhibit GSC proliferation - this effect was suggested to be related with the promotive effect of QUE on differentiation of GSCs into OL-like cells. This is consistent with our previous findings which showed QUE to be unique among the anti-psychiatric drugs based on its promotive effect on cell differentiation: QUE can alleviate pathological demyelination by promoting differentiation of NSCs into myelin-forming OLs [28, 36]. The notion that differentiation of tumor cells is crucial in response to chemotherapy was also supported by other studies [30], Persson et al showed that compared with other glioblastomas, oligodendrogliomas were generated from relatively further differentiated progenitors, and are much more susceptible to conventional chemo-therapies [30]. In agreement with these findings, here we demonstrate that QUE can effectively repress *in vivo* GSCs-initiated tumor growth due to the induction of most of the GSCs to differentiate into OL-like cells.

As DNA methylation and DNA repair are two targeted aspects of antitumor effect for the alkylating chemotherapeutic agent TMZ, the expression of DNA repair enzyme MGMT and removal of methyl residue in GSCs were suggested as underlying the chemo-resistance against TMZ of glioblastomas [33]. We demonstrated that QUE can exert synergistic antitumor effect with TMZ by promoting the differentiation of GSCs into chemotherapy susceptible OL-like cells, thus improving the therapeutic efficiency of TMZ. By treating the cells with QUE after TMZ withdrawal, we demonstrated that QUE can specifically target GSCs and effectively inhibit either TMZ- resistant or –escaped tumors generated from GSCs. Importantly, compared with TMZ's severe cytotoxicity to normal cells, QUE was devoid of cytotoxicity and expressed relatively moderate side effects, such as weight gain and sedation [37]. Since QUE has synergistic antitumor effects with TMZ, combined application may also help reduce treatment dose of TMZ and its related toxic effect. Moreover, the reduced cost of QUE compared to TMZ [38, 39] can be a considerable benefit factor to the patient in chemotherapy of glioma.

Mechanistically, we demonstrated the underlying mechanism for QUE-induced effect on GSC differentiation, i.e. mediation of the inhibition of Wnt signaling pathway. QUE up-regulated phosphorylation of β-catenin and down-regulated p- GSK3β (Ser9), which is closely linked to the regulation of the stemness and differentiation of GSCs [40]. Aberrant activation of the Wnt signaling pathway has been shown to lead to gliomagenesis and tumor growth through nuclear localization of β-catenin [41]. Activation of Wnt signaling also negatively regulates OL lineage development, including specification of OPCs from NSCs and terminal differentiation of OLs [42]. In addition, our previous work showed that selective activation of the Wnt/β-catenin signaling pathway by QS11 can suppress OL differentiation [43]. In line with the aforementioned findings, we showed here that QUE's effect on GSCs can also be attenuated by QS11, a selective agonist of the Wnt/β-catenin signaling pathway; this indicates that QUE-induced bioprocesses in GSCs are likely mediated via the Wnt signaling pathway and suggests a potential application of QUE to induce OL-oriented differentiation for enhanced effectiveness in chemotherapy of glioma.

Differentiation of GSCs is controlled by complicated cell signaling pathways that are activated by different extracellular and intracellular factors, and more intensive studies are required to dissect QUE-induced antitumor effects. For instance, through which up- and/or downstream components does QUE impact the Wnt/β-catenin signaling pathway during GSC differentiation? Previous studies have revealed that QUE is an antagonist of multiple neurotransmitters, which affects activities of dopamine D_1_ and D_2_ receptors, serotonin 5-HT 1A and 2A receptors, and histamine H1 receptor [44]. Notably, inhibition of the dopamine D_2_ receptor can upregulate the phosphorylation of GSK-3β and suppress activation of the Wnt signaling pathway via dishevelled-3 (Dvl-3) [45]. This raises the possibility that the effect of QUE on Wnt signaling may be mediated through dopamine D_2_ activity, though further investigation is required.

Altogether, we demonstrated that QUE can promote the differentiation of GSCs into OL-like cells via inhibition of the Wnt signaling pathway, which led to the sensitization of glioma for TMZ treatment. Therefore, QUE, as an FDA approved drug, emerges as a promising therapeutic candidate for the treatment of malignant glioma.

## MATERIALS AND METHODS

### Glioma stem cell culture

The glioblastoma cell line GL261 was obtained from ATCC (CRL-1887) and maintained in Dulbecco's modified Eagle's medium, DMEM/F12 medium (Gibco, USA) containing 10% fetal bovine serum (FBS) (Gibco, USA), 100 U/ml penicillin G and 100 μg/ml streptomycin (Gibco, USA) for 12 to 18 h. To purify GSCs, the culture medium was first replaced by DMEM/F12 medium with 5% FBS. After 24 and 48 h, the medium was changed to DMEM/F12 medium with 2% or 1% FBS, correspondingly. Lastly, culture medium was replaced by a serum-free medium DMEM/F12 containing 2% B27 (Gibco, USA), recombinant human epidermal growth factor (rhEGF, 10ng/ml; Sigma, USA), and basic fibroblast growth factor (bFGF, 10 ng/ml; Upstate, USA). The procedure above was repeated every 24 h until several primary tumor spheres (namely GSCs) were formed and visible by microscopy. To induce cell differentiation, the tumorospheres (diameter over 200μm) were dissociated by accutase (Sigma, USA) and attached to Poly-D-lysine-coated coverslips and cultured in DMEM/F12 medium containing 10% FBS for up to 7 days.

### Xenografts tumor models and drug treatment

All animal experiments were performed according to an approved protocol from the Laboratory Animal Welfare and Ethics Committee of the Third Military Medical University. In the present study, two GSC-based tumor models were employed. The prepared GSCs (10^4^ cells, in a volume of 10 ul) were either (i) subcutaneously injected into the inguen of nude mice to establish a heterotopic xenograft tumor model, or (ii) orthotopically transplanted into the striatum of C57 mice (coordinate to Bregma: ML 2.00mm, DV 2.20mm, AP 0.14mm) to establish a xenograft glioma mouse model [19]. According to previous study [29], we used 1.5% pentobarbital sodium to anesthetize the mice, and QUE was given to mice 3 h after cell transplantation; at the time the mice were awake and anesthetic drugs were predominantly metabolized and should have minimal (or no) cross-effect on QUE.

To assess the treatment effect of QUE and TMZ, mice were randomly assigned into four groups (n = 10 per group) and treated with: (i) vehicle (VEH, 0.9% NaCl, i.p.) as a control; (ii) QUE (20mg/kg, i.p.)-monotherapy; (iii) TMZ (20mg/kg, i.p.)-monotherapy; or (iv) combined therapy of QUE and TMZ (QUE+TMZ, 20mg/kg of each, i.p.). TMZ and QUE were both dissolved in 0.9% NaCl. For the monotherapies, mice were subjected to daily i.p. injection for 21 days. For the combined treatment of QUE+TMZ, mice were subjected to daily i.p. injection of QUE for 21 days, then, in addition, at day 8, mice started receiving injections of TMZ every other day and until the day of last QUE treatment (Figure 3A). Tumor growth was measured using an external caliper every other day and tumor volume was calculated as length×width^2^/2(mm^2^) [46]. Animals were sacrificed at day 21 after the last drug administration; tumor masses were dissected and harvested for later histological evaluation. To access the specific effect of QUE on GSCs, both the heterotopic xenograft tumor model and the xenograft glioma mouse models were pre-treated with TMZ (20mg/kg, i.p.) for 21 days and then randomly divided into two groups (n = 10 per group). One group was subjected to daily injection of QUE (20mg/kg, i.p.) while the other group was injected with vehicle for 14 days.

### Cell counting assessment

To detect the effect of QUE on GSC proliferation, the number of viable cells was measured using a CCK assay (Dojindo, Japan) according to previously published method [28] with the following modifications. First, cultured tumorospheres were dissociated and single cell suspensions (100μl) were seeded in each of 96-well microplates at a density of 2×10^5^cells/ml. After 12 h incubation, these cells were treated with QUE (dissolved in medium) in gradient concentrations of 0, 1, 5, 10, 20, 50 or 100 μM for up to 48 h. Then, 10 μl of Cell Counting Kit (CCK) solution was added to each well followed by 4 h incubation. Absorbance was measured at 450 nm using a microplate reader (Bio-RAD, Model 680) with a reference wavelength of 650 nm.

### Cell cycle assessment

To examine the effect of QUE on cell division, the cell cycle of the GSCs were analyzed using Flow Cytometry assessment. Briefly, GSCs were incubated in proliferation media with or without 50μM QUE for 48 h. Then the cells were harvested and rinsed with PBS twice and fixed in methanol for 15 mins in a -20°C freezer. After incubation with RNase at 37°C for 30 min, cell nuclei were stained with propidium iodide (PI, 50 μg/ml) for 30 min and examined in a FACS Calibur Flow Cytometer (Becton Dickinson). The cells in different cell cycles (DNA histograms) were analyzed using modified software (Becton Dickinson).

### Bioluminescence imaging

Bioluminescence imaging was used for the detection of tumor growth [47] with modification. Briefly, mice were first anesthetized by inhalation of 2% isoflurane and received intraperitoneal injection of D-luciferin (YEASEN, China) at a dose of 10 ul/g bodyweight. Ten minutes after injection, mice were subjected to image acquisitions in the IVIS Spectrum (Perkin-Elmer) three times at a 2-minute interval. Acquired images were post-processed for quantification using Living Image Software for IVIS Spectrum.

### Immunofluorescence and immunochemistry

Cells grown on coverslips were fixed with 4% paraformaldehyde for immunocytochemistry evaluation according to our previous study [43]. After being incubated with 1% bovine serum albumin (BSA)/0.3% Triton X-100 for 10 min to block non-specific reactions, cells were incubated with primary antibodies (see Table 1) overnight at 4°C followed by an incubation of secondary antibodies (see Table 2) for 2-3 h at room temperature (RT). Lastly, the cells on coverslips were counterstained with DAPI (as a nuclear marker).

**Table 1:** Primary antibodies

Antigen	Source	Dilution	Supplier
CD133	Mouse	1:200 (ICC)	Abcam
Sox2	Rabbit	1:200 (ICC)	Santa Cruz
		1:1000 (WB)	
Nestin	Rabbit	1:200 (ICC/IHC)	Millipore
MBP	Goat	1:200 (ICC/IHC)	Santa Cruz
		1:1000 (WB)	
GFAP	Rabbit	1:400 (ICC/IHC/WB)	Boster
Olig1	Rabbit	1:1000 (WB)	Millipore
Olig2	Rabbit	1:400 (IHC)	Millipore
		1:1000 (WB)	
PCNA	Mouse	1:200 (IHC)	Dako
β-catenin	Rabbit	1:1000 (WB)	Beyotime
P-β-catenin	Mouse	1:1000 (WB)	Santa Cruz
GSK-3β	Rabbit	1:1000 (WB)	Beyotime
pGSK-β	Rabbit	1:1000 (WB)	Beyotime
β-actin	Mouse	1:1000 (WB)	Santa Cruz

**Table 2:** Secondary antibodies

Antibody	Dilution	Supplier
Anti-rabbit-TRITC	1:1000	Santa Cruz
Anti-rabbit- FITC	1:1000	Santa Cruz
Anti-mouse-TRITC	1:1000	Santa Cruz
Anti-goat-FITC	1:1000	Santa Cruz
Anti-rabbit-HRP	1:1000	Santa Cruz
Anti-goat-HRP	1:1000	Santa Cruz
Anti-mouse-HRP	1:1000	Santa Cruz

For histological evaluation, tumor tissues were sliced (5μm thickness) and either stained with hematoxylin and eosin (H.E. staining), or used for immunohistochemistry staining [29]. Briefly, the slides were first blocked with PBS containing 0.8% hydrogen peroxide or PBS with 0.3% Tween-20 and 5% BSA, respective to stain protocol, to reduce endogenous peroxidase activity and non-specific reaction, then incubated with the primary antibodies (see Table 1) for 12 h at 4°C, followed by an incubation with second antibody (see Table 2) for 5 h at RT. Lastly, the antigen-antibody complexes were visualized using DAB (Boster, China) as the chromogen.

### Image acquisition and quantification

For immunofluorescence staining, immuno-reactivity was determined by a confocal laser-scanning microscope (PV100, Olympus) with excitation wavelengths appropriate for FITC (488 nm), Cy3 (552 nm) or Cy5 (625 nm). Immunochemical staining results were determined by microscope (BX60, Olympus), with at least three samples selected from each group for observation. For each section at least 9 fields were chosen under the scope. Immunofluorescence positive cells and optical density were counted by Image-Plus Pro software 5.0 (Media Cybernetics, Silver Spring, MD, USA).

### Western blot analysis

Proteins were extracted from cultured cells and tumor tissues using RIPA lysis buffer with freshly supplemented 1% PMSF solution (Biocolors, China). Cell lysates, each containing 50 μg total protein, were separated through 12% SDS-PAGE gels and transferred to polyvinyldifluoride membranes. For immuno-blotting, membranes were first blocked in PBST (with 5% non-fat milk) for 1 h at RT, then incubated with the primary antibodies for 24 - 48 h at 4 °C, followed by a incubation with secondary antibodies for 2 h at RT (see Table 1 and 2). Anti-β-actin was used as the loading control. Immunoreactive bands were visualized using an ECL plus detection kit (Amersham Biosciences, Buckinghamshire, UK), and developed using Image station 4000 (Kodak Carestream). All Western blot results were analyzed by semiquantitative analysis using Image-Pro Plus 6.0 (Media Cybermetics, Rockville, MD, USA).

### Statistical analysis

Data were presented as mean ± standard error of the mean (SEM). All quantitative data were analyzed by one- or two-way analysis of variance (ANOVA), followed by Tukey's multiple comparisons using GraphPad Prism (GraphPad, USA) software. A p-value less than 0.05 was considered a significant difference.

## SUPPLEMENTARY MATERIALS FIGURES


