# Supplementary material for: Promoting oligodendroglial-oriented differentiation of glioma stem cell: a repurposing of quetiapine for the treatment of malignant glioma
Source: Oncotarget. 2017 Mar 21;8(23):37511–24. doi: 10.18632/oncotarget.16400 (PMC5514926; doi:10.18632/oncotarget.16400)
Supplement: Supplementary file 1 [file oncotarget-08-37511-s001.pdf]

Promoting oligodendroglial-oriented differentiation of glioma stem cell: A repurposing of quetiapine for the treatment of malignant glioma

SUPPLEMENTARY FIGURES

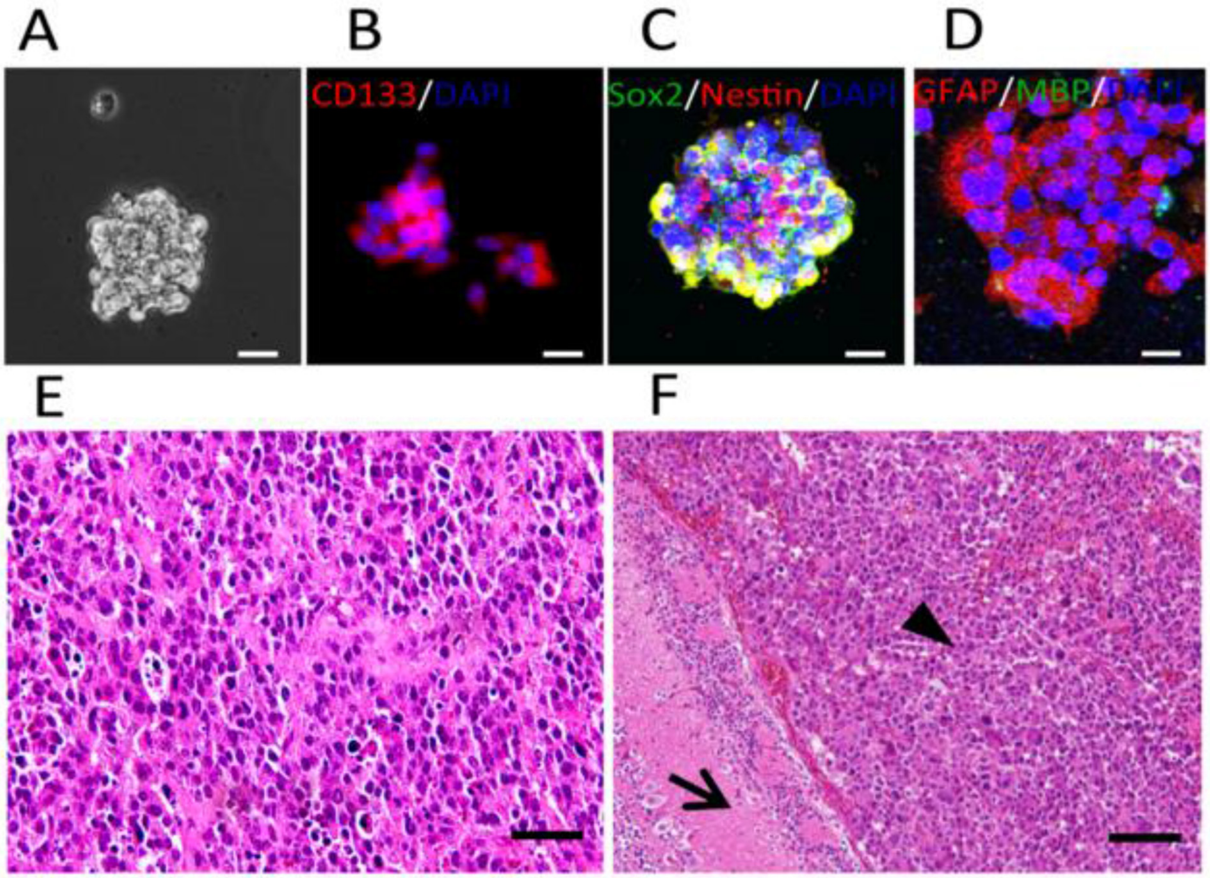

Supplementary Figure 1: Identification of GSCs purified from glioblastoma cell line GL261. **(A)** Representative cellular image of tumor sphere formation under phase contrast microscope. **(B-D)** Representative images of immunofluorescence staining showing GSCs expressing lineage markers CD133 in red **(B)**, Sox2 in green and Nestin in red **(C)**, GFAP in red and MBP in green **(D)**; DAPI in blue **(B-D)**. **(E)** Representative picture of hematoxylin and eosin (HE) staining of subcutaneous GSCs-initiated xenograft tumor; **(F)** Representative picture of HE staining of GSC-initiated orthotopic xenograft glioma. Arrow: normal brain tissue; filled triangular arrow head: tumor tissue. Scale bar = 20 µm **(A-D)**, 50µm **(E)**, and 100µm **(F)**.

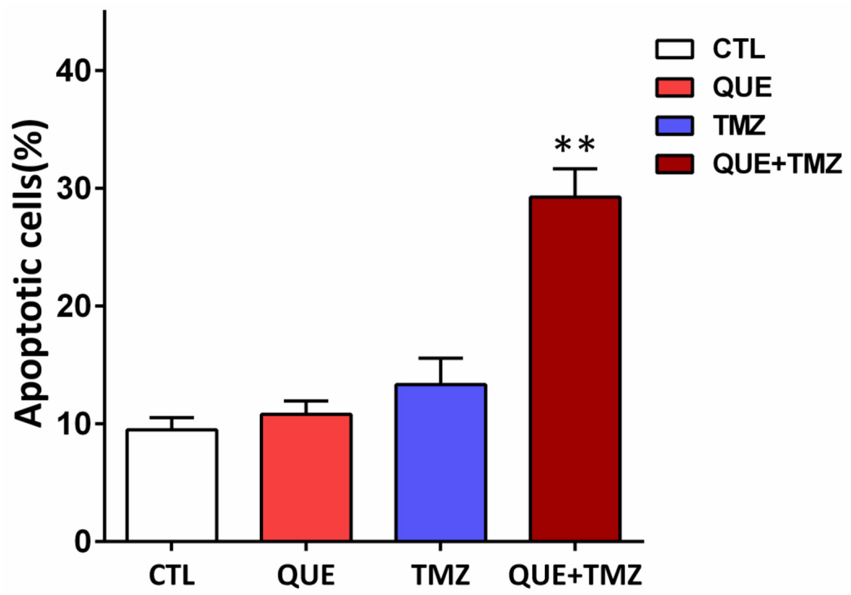

Supplementary Figure 2: Characterization of cell apoptosis after drug treatment. Quantitative analysis for apoptotic cells by Annexin V/PI staining after 48 h treatment of vehicle control (CTL), QUE, TMZ, or QUE+TMZ. **P < 0.01 *vs* other groups.
